# Transmission of fungal partners to incipient *Cecropia*-tree ant colonies

**DOI:** 10.1371/journal.pone.0192207

**Published:** 2018-02-21

**Authors:** Veronika E. Mayer, Maximilian Nepel, Rumsais Blatrix, Felix B. Oberhauser, Konrad Fiedler, Jürg Schönenberger, Hermann Voglmayr

**Affiliations:** 1 Department of Botany and Biodiversity Research, University of Vienna, Rennweg 14, Vienna, Austria; 2 Department of Microbiology and Ecosystem Science, University of Vienna, Althanstraße 14, Vienna, Austria; 3 Centre d’Ecologie Fonctionnelle et Evolutive, CNRS UMR 5175, France; 4 Department of Zoology, University of Regensburg, Universitätsstraße 31, Regensburg, Germany; Indian Institute of Science, INDIA

## Abstract

Ascomycete fungi in the nests of ants inhabiting plants (= myrmecophytes) are very often cultivated by the ants in small patches and used as food source. Where these fungi come from is not known yet. Two scenarios of fungus recruitment are possible: (1) random infection through spores or hyphal fragments from the environment, or (2) transmission from mother to daughter colonies by the foundress queen. It is also not known at which stage of the colony life cycle fungiculture is initiated, and whether the- symbiont fungi serve as food for the ant queen. To clarify these questions, we investigated four *Azteca* ant species inhabiting three different *Cecropia* species (*C*. *insignis*, *C*. *obtusifolia*, and *C*. *peltata*). We analysed an rRNA gene fragment from 52 fungal patches produced by founding queens and compared them with those from established *Azteca* colonies (n = 54). The infrabuccal pockets of winged queens were dissected to investigate whether young queens carry fungi from their mother colony. Additionally, ^15^N labelling experiments were done to verify whether the queen feeds on the patches until she is nourished by her first worker offspring. We infer from the results that the fungi cultivated in hollow plant structures are transferred from the parental colony of the young queen. First, fungal genotypes/OTU diversity was not significantly different between foundress queen patches and established colonies, and second, hyphal parts were discovered in the infrabuccal pockets of female alates. We could show that fungiculture already starts before queens lay their eggs, and that the queens do not feed on fungal patch material but feed it to the larvae. Our findings suggest that fungiculture may be crucial for successful colony founding of arboreal ants in the tropics.

## Introduction

Fungus farming in leaf cutter ants is famous; they grow basidiomycetes for food and manure the fungal gardens with various substrates. In other ant taxa, fungi have occasionally been reported to be present in the nests, but are generally regarded to be pathogens or merely commensals [1–7], or have simply remained overlooked as they form inconspicuously small dark patches inside the hollow stems, petioles, or leaf pouches (so-called “domatium”) of the host plants (myrmecophytes). Only in recent years has it become evident that these fungi are not accidental inhabitants of the ant nests and are actively cultivated and used by ants [8, 9], and represent a third symbiotic partner in many tropical ant-plant mutualisms [10, 11].

Most fungi cultivated inside the domatium belong to the order *Chaetothyriales* (Ascomycota), a group of”black yeasts” characterized by slow growing and melanized hyphae. *Chaetothyriales* are highly diverse with respect to their lifestyles. They may be endophytes in plant tissues [12], plant pathogens [13], epiphytes colonizing leaves of tropical trees [14], rock colonizers in extreme habitats [15], or colonizers in human-made habitats like washing machines, dish washers, or metro tunnels [16, 17]. Some are pathogens and are found to be infecting animal and human skin and central nervous system [18]. Ants are the only animals known so far to live in a mutualistic relationship with chaetothyrialean fungi.

During the past couple of years, about 17 ant-plant associations that involve obligate chaetothyrialean fungi have been described [10, 11]. In these mutualistic associations, fungi are used for nest construction, sophisticated prey capture and defence mechanisms [19–22]. They are grown in small patches in the domatium and fed to the larvae [9, 23–25]. Some fungal strains isolated from domatia are ubiquitous and not specific to either the ant or the plant species, whereas other strains have been found to show some degree of ant-host specificity [23, 26].

Thus far, it is unknown how fungus-ant associations become established during the colony life cycle of obligate plant-ants. Until now, ant associated *Chaetothyriales* were described only from established colonies [5, 9–11, 23, 24]. When and how fungiculture is started, whether the queen carries the fungi along from her mother colony, transmitting the symbiont vertically; or whether inoculation occurs haphazardly when the young queen enters the domatium cavity or even later during patrolling of the worker ants (horizontal transmission), is so far not described from any ant-plant system with fungiculture.

To address these questions, we investigated fungiculture by *Azteca* queens founding new colonies in young *Cecropia* trees (Urticaceae). In the genus *Cecropia*, a group of neotropical pioneer trees, 46 of the 61 species are associated with ants [27]. A recent multigene phylogeny inferred a single origin of the symbiotic relationship between *Azteca* ants and *Cecropia* plants, starting around 8 Mya ago [28]. The *Cecropia* hosts provide hollow stem internodes for housing (domatia) and phyto-glycogen containing food bodies (Müllerian bodies) [29] for nutrition. The ants in return deter herbivores, prune their host trees from encroaching vegetation, and deposit extra nutrients within the hollow stem where they may be absorbed into the host tree's tissue [30–32]. In hollow stem internodes of *Cecropia* inhabited by *Azteca* colonies, we regularly found chaetothyrialean fungi in small, clearly delimited patches. Some of the fungus strains found were shared among different *Azteca* species, while others were ant-species specific [23]. This pattern indicates two possible scenarios of fungus recruitment: (1) random infection through spores or hyphal fragments from the environment, or (2) transmission from mother to daughter colony by the foundress queen. If foundress queens carry along fungi from their mother colonies, the distribution and frequency of fungal strains in foundress queen colonized domatia should show the same pattern as observed in established colonies. If no such pattern is observable, the inoculation is suggested to originate from random infection.

In the present study, we observed the queens’ behaviour during colony foundation in the field, determined the fungal strains in patches of *Azteca* foundress queens with molecular methods, and compared the pattern of fungal strains with that of established *Azteca* colonies from the same sampling sites. We morphologically examined the infrabuccal pockets of alates before and after their nuptial flight for presence of fungi. Finally, we investigated the role of fungi as possible food source for the queen during the claustral colony founding (a stage in which queens do not forage but seal their nest during colony founding) using stable isotope analysis.

## Material and methods

### Study sites and species identification

Observations and sample collections were made in SW Costa Rica near the Tropical Research Station La Gamba (www.lagamba.at; N08°42’03”, W083°12’06”, 70 m asl) and near the Monteverde cloud forest between Guacimál (N10°12’57”, W084°50’46”) and Santa Elena (N10°19’12”, W084°49’30”) (345–1448 m asl). Patches of foundress queens and established colonies were sampled at the same sites. The *Cecropia* trees with colony founding *Azteca* queens were *C*. *insignis* Liebm., *C*. *obtusifolia* Bertol., and *C*. *peltata* L., between 0.5–2 m tall with a diameter of 1–3 cm, and grew along roadsides or in forest gaps. Ants (*Azteca alfari* Emery, *A*. *coeruleipennis* Emery, *A*. *constructor* Emery, *A*. *xanthochroa* Roger) were identified with the key provided by Longino [33].

### Foundress queens’ behaviour

A foundress queen was defined as a non-physogastric dealate mated female observed in a recently colonised domatium, without any open entrance holes. Foundress queens either had no associated brood, eggs only, or brood at all stages; some were accompanied by a few dwarf workers.

We opened the stems of 64 young *Cecropia* plants (17 from Monteverde, 47 from La Gamba; in total 180 domatia with 212 living queens; see S2 Table), and documented the following parameters: the number of foundress queens and *Azteca* species per domatium (sometimes more than one species), the condition of the parenchyma on the inner domatia wall, the presence or absence of fungal patches, the presence or absence of eggs, larvae, pupae, and, in a few cases, newly hatched dwarf workers. Twice we were able to film a recently dealate gyne while biting open, entering through, and subsequently sealing the entrance hole to the domatium cavity with parenchyma scraped from the domatium wall (S1A–S1E Fig).

### Fungal patch collection, DNA sequencing and analysis

Only fungal patches from domatia with a sealed entrance hole (see S1E Fig), which were big enough (diameter >3 mm) for sufficient yield of fungal DNA, were considered for molecular analysis (S1A and S1B Table). Whole patches were collected with sterile forceps, placed in plastic vials, closed with air-permeable cotton wool, and dried in a box with silica gel. In all but one case, we found only one patch per domatium, even though colony founding by multiple foundresses in one domatium (pleometrosis) was quite common (S2A Fig).

In total, 52 fungal samples from small piles made by the foundress queens (in the following named “foundress patches”) were sampled for sequencing. The majority (n = 45) were individual foundress patches with eighty-seven queens involved (12 *Azteca alfari*, 7 *A*. *coeruleipennis*, 33 *A*. *constructor*, 35 *A*. *xanthochroa*). In one domatium colonized by an *A*. *coeruleipennis* queen, two fungal patches occurred. These were separately collected and analyzed. Six samples were pooled from several rather small patches of *A*. *alfari* queens (S1A Table). Parenchyma from the inner wall of five uninhabited domatia was collected as control. In addition, 25 patch samples from established *Azteca* ant colonies defined by a distinct worker caste and re-opened domatia entrances were also analysed.

DNA extraction, PCR, and Sanger dideoxy sequencing was performed according to the protocols published earlier [10, 23]. For identification, we used the ITS1-5.8S-ITS2 (ITS) rRNA gene; details on primers, PCR and sequencing are described in [23]. Mixed sequences were identified using a sequence comparison approach by comparing ambiguous alignment positions with the sequences of known genotypes as described earlier [23]. All sequences are deposited in GenBank (http://www.ncbi.nlm.nih.gov/genbank/). Strains and GenBank accession numbers from this study are listed in S1A and S1B Table.

A matrix containing new sequences from the current study, representative sequences of *Azteca* associated operational taxonomic units (OTUs) [23] and selected sequences from other fungi representing the “domatia symbiont clade” [10] was aligned using Muscle version 3.8.31 [34] and checked with BioEdit version 7.2.5. [35]. The final data matrix for the analysis contained 468 nucleotide positions from 78 nucleotide sequences.

For phylogenetic reconstruction, a maximum likelihood analysis was done using MEGA7 [36], based on the Kimura 2-parameter (K2) model [37] with a discrete Gamma distribution (+G; 5 categories, parameter = 0.4209) and a proportion of evolutionarily invariable sites (+I; 41.37% sites). Initial trees for the heuristic search were obtained by applying the Neighbour-Joining method to a matrix of pairwise distances estimated using the Maximum Composite Likelihood (MCL) approach; heuristic search was done with extensive subtree-pruning-regrafting (SPR level 5) branch swapping.

Trees were rooted with *Cladophialophora scillae* [EU035412] and *Cladophialophora hostae* [EU035407] as outgroups.

### Infrabuccal pocket content

From four female alates (two *A*. *constructor*, one *A*. *alfari* and *A*. *coeruleipennis*) infrabuccal pockets were investigated. After dissecting the heads, the infrabuccal pocket content was put on a glass slide and stained with 10 μL of calcofluor white M2R (1 g/L) (Sigma-Aldrich Co., USA) according to the manufacturers guidelines. Calcofluor white is a non-specific fluorochrome that binds to cellulose and chitin in cell walls. The samples were investigated with an epifluorescence microscope (Zeiss Axio Imager.M1) using UV excitation of 365 nm.

### ^15^N labelling of foundress patches

A small “window” was cut into domatia with sealed entrance holes, 4 μl of a 98 at% ^15^N amino acid mixture (Isotec Sigma-Aldrich, USA) were pipetted with a Hamilton syringe in four 1 μl droplets directly into the patch pile. After each droplet we waited for two minutes to be sure that the liquid was completely imbibed from the patch substrate and not scattered over the surface. Thereafter, the window was sealed again using duct tape. After 7 days of incubation, patches (n = 5), queens (n = 5), brood (larvae: n = 4, pupae: n = 5) and workers (n = 3) from domatia with queens still alive and free of mould were collected and dried. Queen (and–if present also the workers) were analysed only after having removed their legs to be sure that possible contamination on the tarsal did not adulterate the result. The larvae and pupae are not moving and the measured ^15^N must result from the consumption of fungal patch particles. The dried samples were weighed into tin capsules for isotope ratio mass spectrometric analysis (IRMS) in a continuous-flow IRMS system that consisted of an elemental analyser (EA 1110, CE Instruments, Milan, Italy) connected to an IRMS (DeltaPLUS, Finnigan MAT, Bremen, Germany) by a ConFlo II interface (for details see [38]). Samples of non-incubated patches (n = 13), queens (n = 5), larvae (n = 7), pupae (n = 7) and workers (n = 5) served as natural abundance controls.

### Statistics

With contingency tables, we tested (a) whether the distribution pattern of fungal OTUs is the same between foundress queen and established colony patches, and (b) whether significantly more OTUs occur if more than one queen is present in the same domatium. Due to the small sample numbers, Fisher’s exact test was used. In one exception, where all samples were pooled, a Monte Carlo Chi^2^ was applied [39].

## Results

### Behaviour of foundress queens

Based on the observation of 180 domatia inhabited by 212 queens from 64 young *Cecropia* plants, we found internodes 2–6 (counted from the apex) preferably colonised. Queen number per plant ranged between 1 and 16 (data not shown) and was much higher in Monteverde with on average 6.7 (ranging from 1–16) foundress queens per plant compared to 1.9 (range 1–4) in La Gamba (S2 Table).

**Fig 1:**
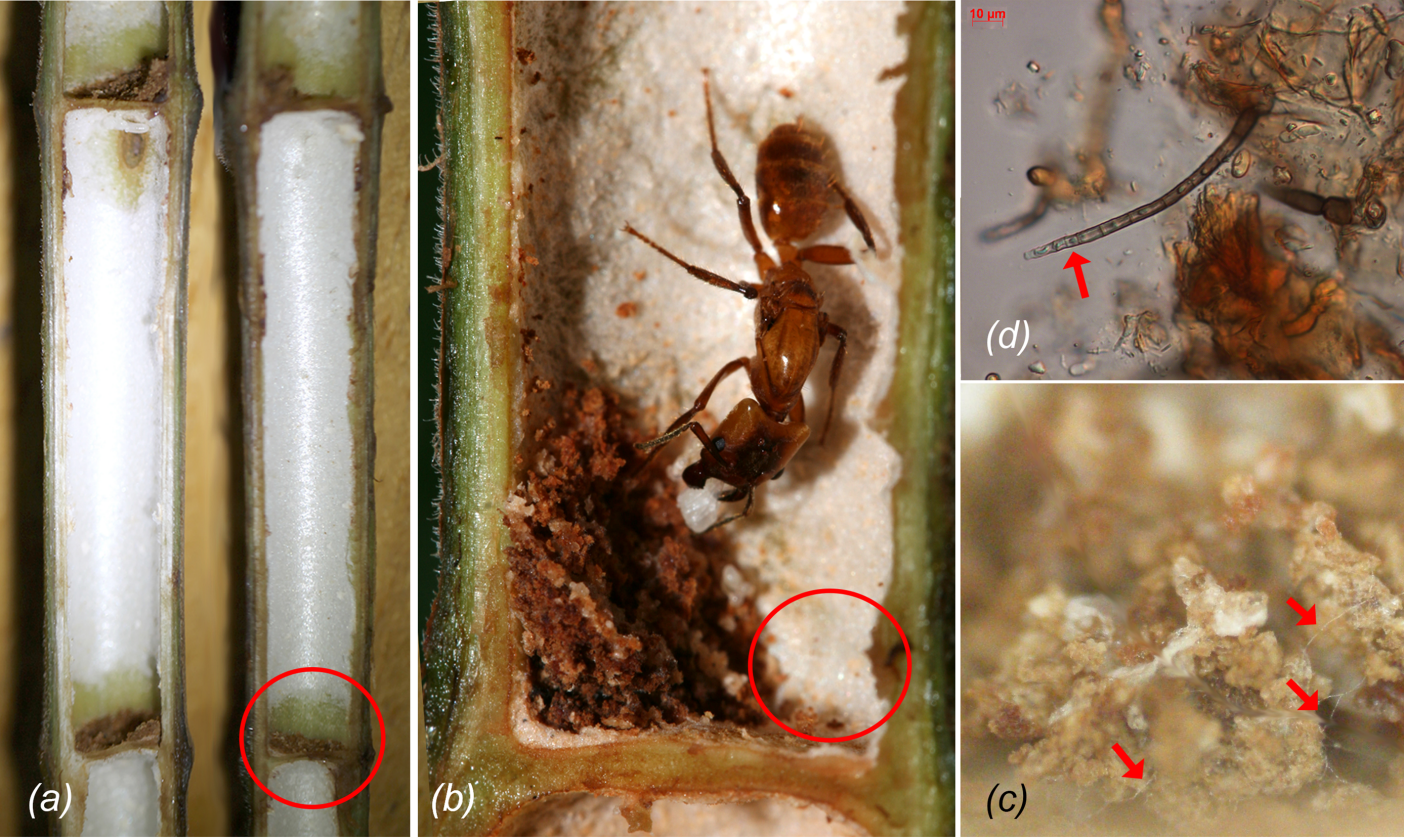
Colonization of a *Cecropia* sp. domatium. *(a)* Parenchyma (white tissue) of the inner domatium wall. The part from where it has been scraped off is marked with a circle; *(b)* An *Azteca xanthochroa* queen with a parenchyma pile inoculated with chaetothyrialean fungi (foundress patch). Eggs and larvae are deposited next to the fungal patch (circle); *(c)* Detail of an *Azteca constructor* foundress patch with hyphae and *(d)* conidiophores (arrowheads). Scale bars: *(a)* 2cm, *(b)* 2mm.

During initial colonization, queens not only started to immediately scrape the spongy white parenchyma from the inner domatia walls to seal the entrance hole (S1C–S1E Fig), but also amassed parenchyma into a small pile (the “foundress patch”) of 2–10 mm in diameter (Fig 1A and 1B). Even in very young plants with little developed parenchyma, pile-making was a priority task of the queens (S2B Fig). Eggs and larvae were only observed in domatia with such a pile of parenchyma tissue and they were usually deposited next to it (Fig 1B).

### Fungal OTUs in foundress patches compared to established colonies

**Fig 2:**
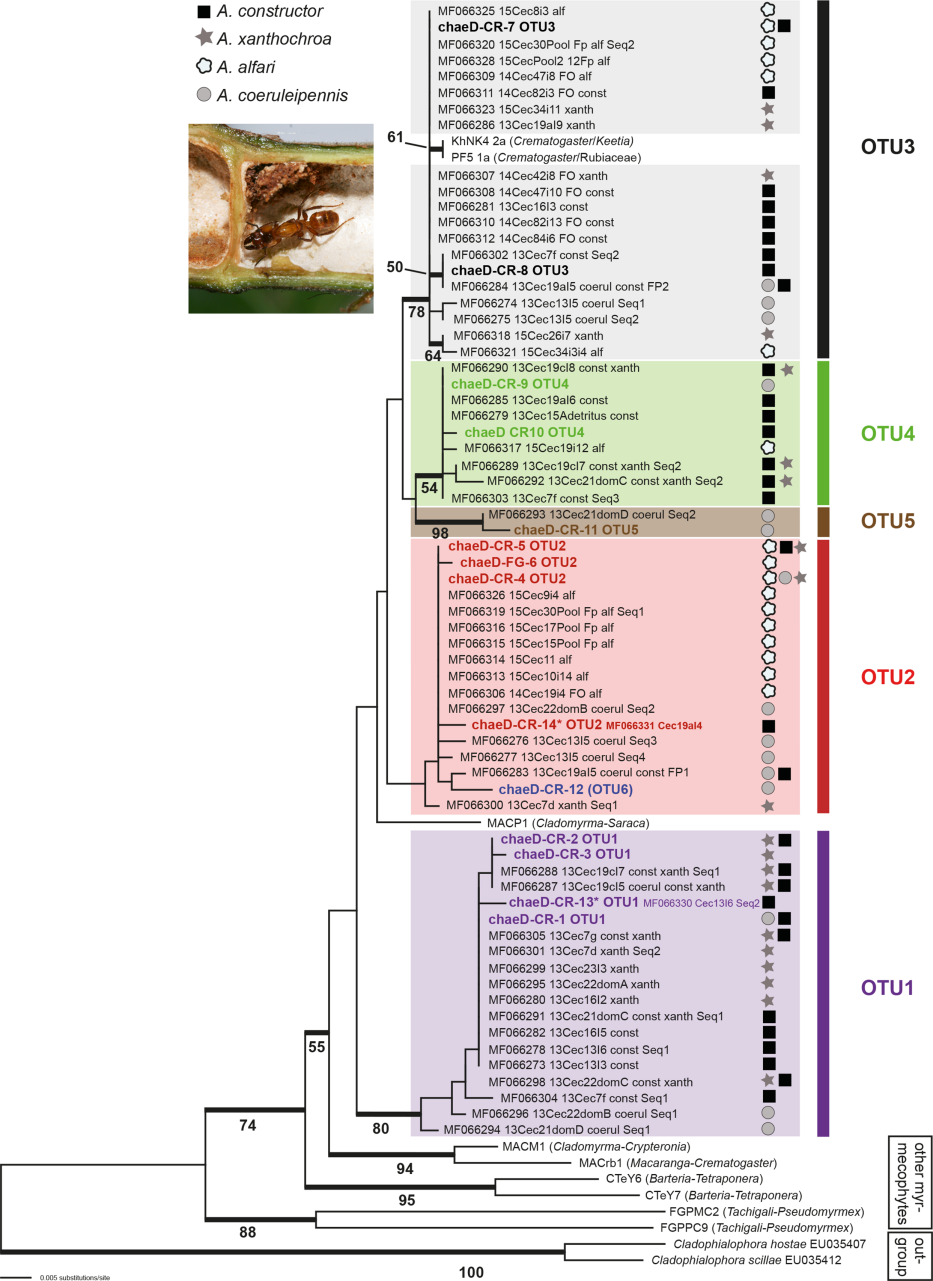
Molecular phylogenetic analysis of fungal genotypes associated with patches from *Azteca* foundress queens. A maximum likelihood analysis of the ITS matrix was performed with MEGA7 [36], showing the best tree (-lnL = 1906.92) based on the Kimura 2-parameter (K2) model [37], applying a discrete Gamma distribution (+G; 5 categories, parameter = 0.4209) and a proportion of evolutionarily invariable sites (+I; 41.37% sites). The tree is drawn to scale, with branch lengths measured in the number of substitutions per site. Bold branches indicate ML bootstrap support above 50%.

From parenchyma samples of uninhabited internodes we could not amplify any chaetothyrialean DNA. However, chaetothyrialean DNA was invariably found in foundress patches (Fig 1C and 1D). From 52 samples we got 64 different fungal sequences that could be assigned to 14 different genotypes and five OTUs (Fig 2; S1 Table). Nine samples contained more than one genotype. OTU1 was the most common one in foundress patches, followed by OTU3 and OTU2. OTU1 was not found in any of the *A*. *alfari* patches; instead OTU2 occurred in at least one foundress patch of each ant species (Table 1). With the extended sequence data, OTU6 from an earlier study [23] appeared within the OTU2 cluster and is no longer supported as a separate clade.

**Table 1:** Comparison of genotype and OTU (operational taxonomic unit) occurrence in foundress queen patches (FP n = 52) and patches of established colonies (EP n = 54). The number represents how often the genotype was found in the respective ant species (in %) relative to all samples of the respective ant species. In nine patches, more than one genotype was detected (n = 9 for queen and established patches). In eleven domatia, more than one founding queen occurred (up to 10). Genotype frequency is shown for each *Azteca* species separately. In some cases, the percentage exceeds 100% due to patches with more than one fungal OTU.

genotype	genotype frequency [%]		*A*. *alfari*		*A*. *coerul*		*A*. *const*		*A*. *xanth*	
	FP all n = 52	EP all n = 54	FP n = 13	EP n = 22	FP n = 7	EP n = 3	FP n = 22	EP n = 24	FP n = 18	EP n = 5
**OTU1**	**39.0**	**18.5**	**-**	**4.6**	**42.9**	**33.3**	**47.6**	**16.7**	**55.6**	**100.0**
chaeD-CR-1	27.1	14.8	-	4.6	14.3	33.3	35.0	16.7	44.0	40.0
chaeD-CR-13	1.7	-	-	-	-	-	4.8	-	-	-
chaeD-CR-2	8.5	1.9	-	-	28.6	-	9.5	-	11.1	20.0
chaeD-CR-3	-	1.9	-	-	-	-	-	-	-	40.0
**OTU2**	**30.5**	**53.7**	**61.5**	**95.5**	**71.4**	**100.0**	**14.3**	**16.7**	**11.1**	**40.0**
chaeD-CR-5	20.3	37.0	61.5	68.2	14.3	33.3	4.8	16.7	11.1	-
chaeD-CR-4	6.8	13.0	-	13.6	42.9	66.7	15.0	-	-	40.0
chaeD-CR-14	5.1	-	-		-	-	4.8	-	-	-
chaeD-FG-6	-	1.9	-	13.6	-	-	-	-	-	-
chaeD-CR-12	-	1.9	-		-	-	-	-	-	-
**OTU3**	**39.0**	**46.3**	**38.5**	**13.6**	**42.9**	**33.3**	**38.1**	**75.0**	**38.8**	**60.0**
chaeD-CR-8	5.1	3.7	-	-	28.6	-	4.8	8.3	-	-
chaeD-CR-7	33.9	42.6	38.5	13.6	14.3	33.3	33.3	66.7	38.8	60.0
**OTU4**	**15.3**	**9.3**	**7.7**	**-**	**14.3**	-	**23.8**	**16.7**	**11.1**	
chaeD-CR-9	15.3	1.9	7.7	-	14.3	-	23.8	4.2	11.1	-
chaeD-CR-10	-	7.4	-	-	-	-	-	12.5	-	-
**OTU5**	**1.7**	**1.9**	**-**	**-**	**14.3**	**33.3**	**-**	**-**	**-**	**-**
chaeD-CR-11	1.7	1.9	-	-	14.3	33.3	-	-	-	-

OTU distribution was not significantly different between foundress queens and established colonies for patches from domatia with single queens (Fisherʼs *p* = 0.666). However, if in addition to single queen domatia also those containing multiple queens were considered, a significant difference was found (Monte Carlo Chi^2^ *p* = 0.0153).

At the level of individual ant species, the OTU distribution was not significantly different between foundress queens and established colonies. In both cases *A*. *alfari* and *A*. *coeruleipennis* cultivated more often OTU2, *A*. *xanthochroa* OTU1, and *A*. *constructor* OTU1 and 3 (Table 1). OTU5 occurred only in *A*. *coeruleipennis* (14% of all patches).

### Number and diversity of foundress queens and the range of fungal OTUs

**Fig 3:**
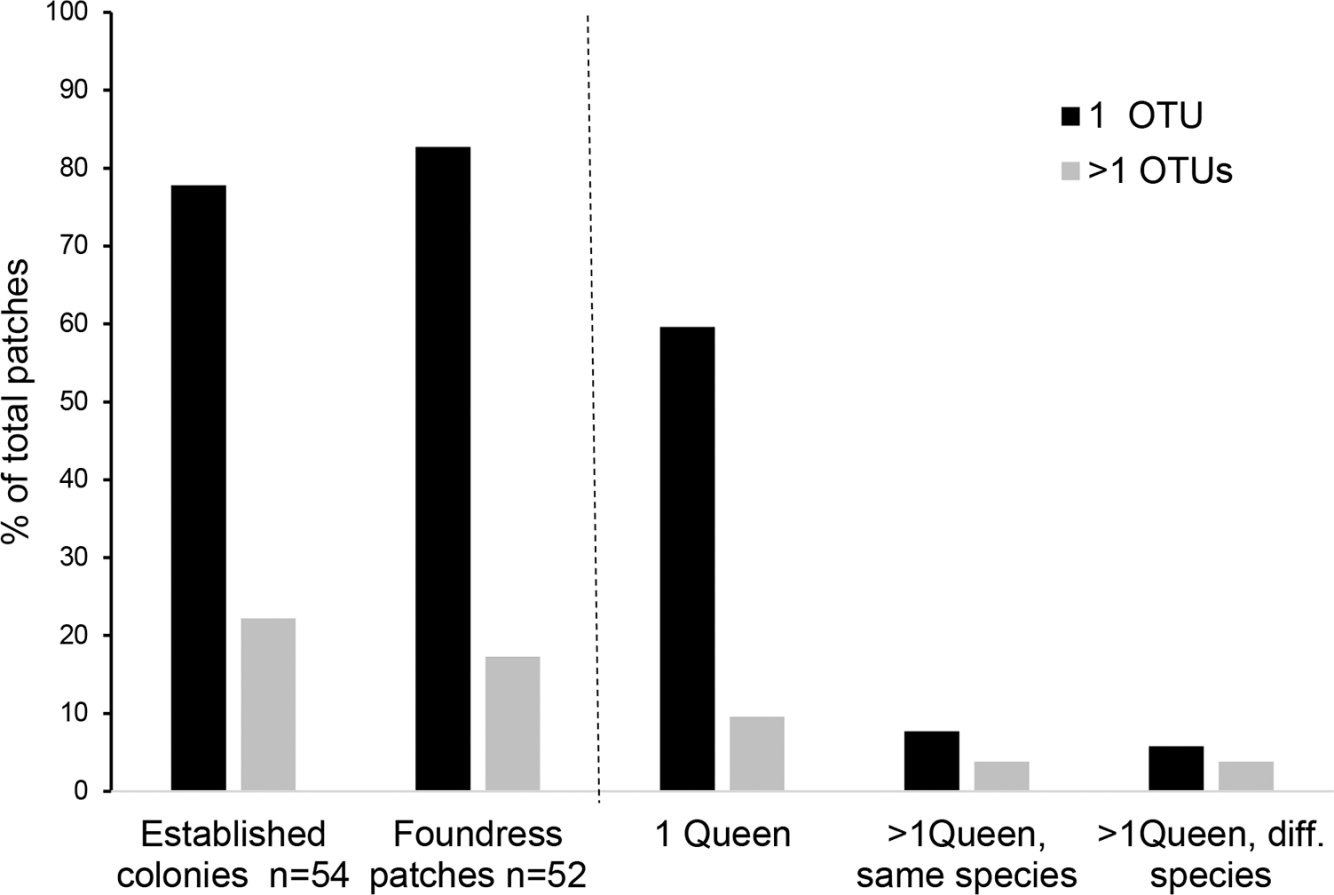
Frequency of patches with one or multiple fungal OTUs in foundress queen patches compared to established colonies. In domatia with a single queen patches with only one OTU is common, whereas in multiqueen domatia with several queens of the same species as well as of different species multi-OTU patches are equal to those with only one OTU.

At the founding stage, single-OTU patches (43) dominated clearly over multi-OTU ones (9), also in established colonies (43 single-OTU, 12 multi-OTU) (Table 2; Fig 3). Interestingly, five multi-OTU patches were from domatia with only one queen, and four from domatia with more than one queen. Twelve patches had only one fungal OTU although they were colonised with more than one queen—six of them even with queens from different *Azteca* species (Fig 3; Table 2). In the majority of cases, only one foundress queen was observed, and multiple queens occurred in 21.7% of the domatia in Monteverde and in 3% in La Gamba. They were either from the same species (up to 10 *A*. *xanthochroa* females in one domatium), or from different *Azteca* species (e.g., *A*. *xanthochroa* and *A*. *constructor*, *A*. *coeruleipennis* and *A*. *constructor*) (S2 Table).

**Table 2:** Number of OTUs in patches of individual established colonies compared with patches of foundress queens. For foundress queen samples, it is indicated whether we found (i) a single queen (1 Q), (ii) more than one of the same *Azteca* species (>1 Q same sp.) or (ii) more queens of different *Azteca* species (>1 Q diff spp.) in one domatium.

	Established n = 54	Foundress n = 52	Foundress queens/domatium		
			1 Q	>1Q same sp.	>1Q diff. spp.
**all samples**					
1 OTU	42	43	31	6	6
>1 OTUs	12	9	5	2	2
***alfari***					
1 OTU	19	14	13	0	1
>1 OTUs	3	1	0	1	0
***coeruleipennis***					
1 OTU	0	4	3	0	1
>1 OTUs	3	3	3	0	0
***constructor***					
1 OTU	21	18	11	2	5
>1 OTUs	3	4	1	1	2
***xanthochroa***					
1 OTU	2	14	8	0	6
>1 OTUs	3	3	0	1	2

A higher number of OTUs in the fungal patches was not related to the presence of more than one queen per domatium (Fisher's *p* = 0.618); this result remained robust in conspecific as well as mixed species groups for all four *Azteca* species. At the ant species level multi-OTU patches were significantly more often found in multi-queen domatia with *A*. *xanthochroa* involved (Fisher's *p =* 0.0458). For *A*. *constructor* (Fisher's *p =* 0.237) and *A*. *coeruleipennis* (Fisher's *p =* 0.4286) the relationship was not significant. *A*. *alfari* had single OTUs with only one exception. In contrast, most *A*. *coeruleipennis* patches with one queen had more than two fungal OTUs (Table 2).

### Infrabuccal pocket content

**Fig 4:**
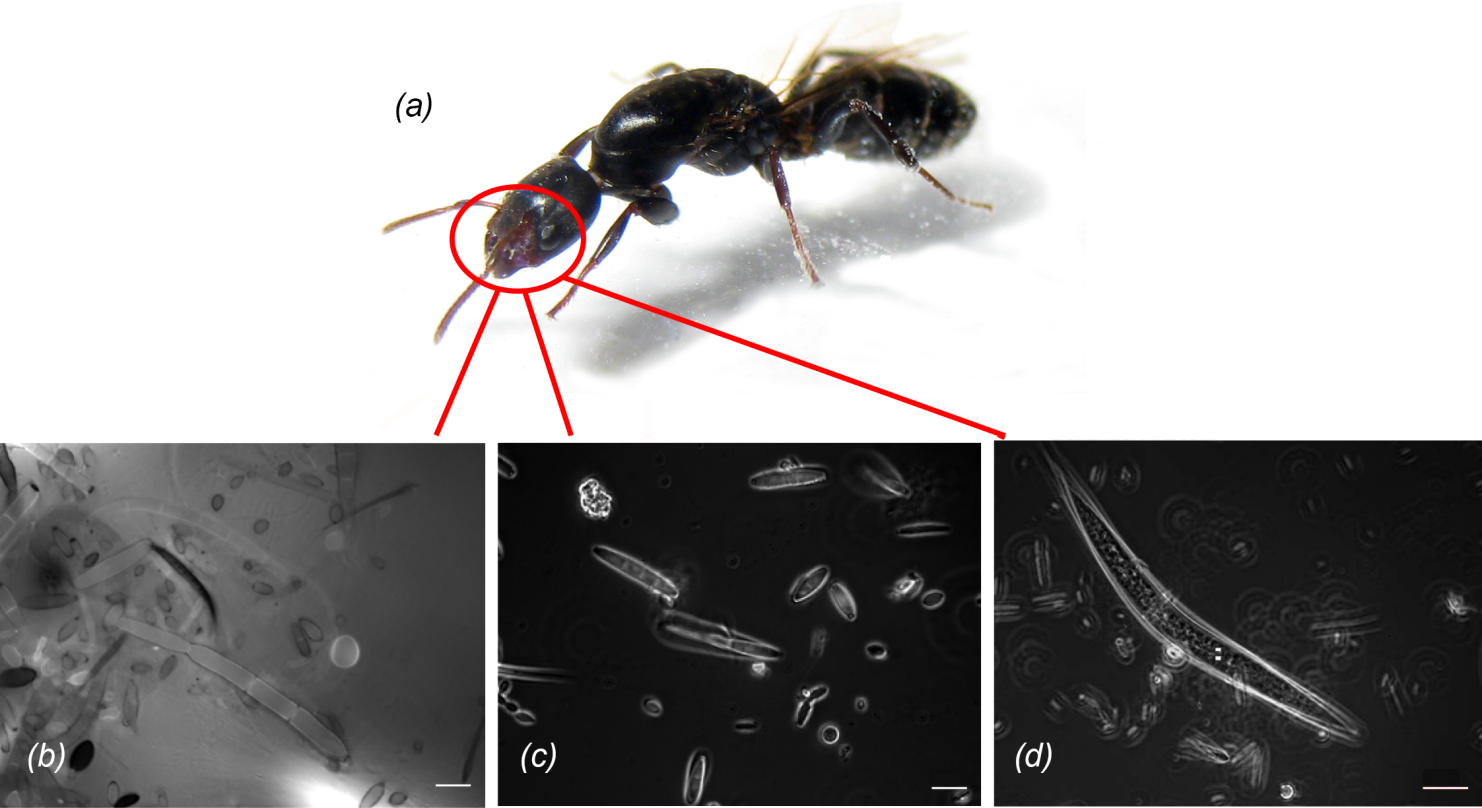
Infrabuccal pocket content of founding queens. Founding queens *(a)* carry in their infrabuccal pockets *(b)* hyphae, *(c)* a number of unclassified particles, and *(d)* nematode dauerlarvae. Infrabuccal pocket content was stained with calcofluor white and investigated with an excitation of 365 nm *(b*, *c)* and brightfield *(d)*. Scale bars: *(b*, *c)* 10μm, *(d)* 20μm.

In their infrabuccal pockets, alates carried hyphal fragments that had the same morphology and size as the fungi in the foundress patches. Additionally, spores, nematodes, nematode eggs, and many other unidentified particles were found (Fig 4).

### ^15^N labelling of foundress patches

**Fig 5:**
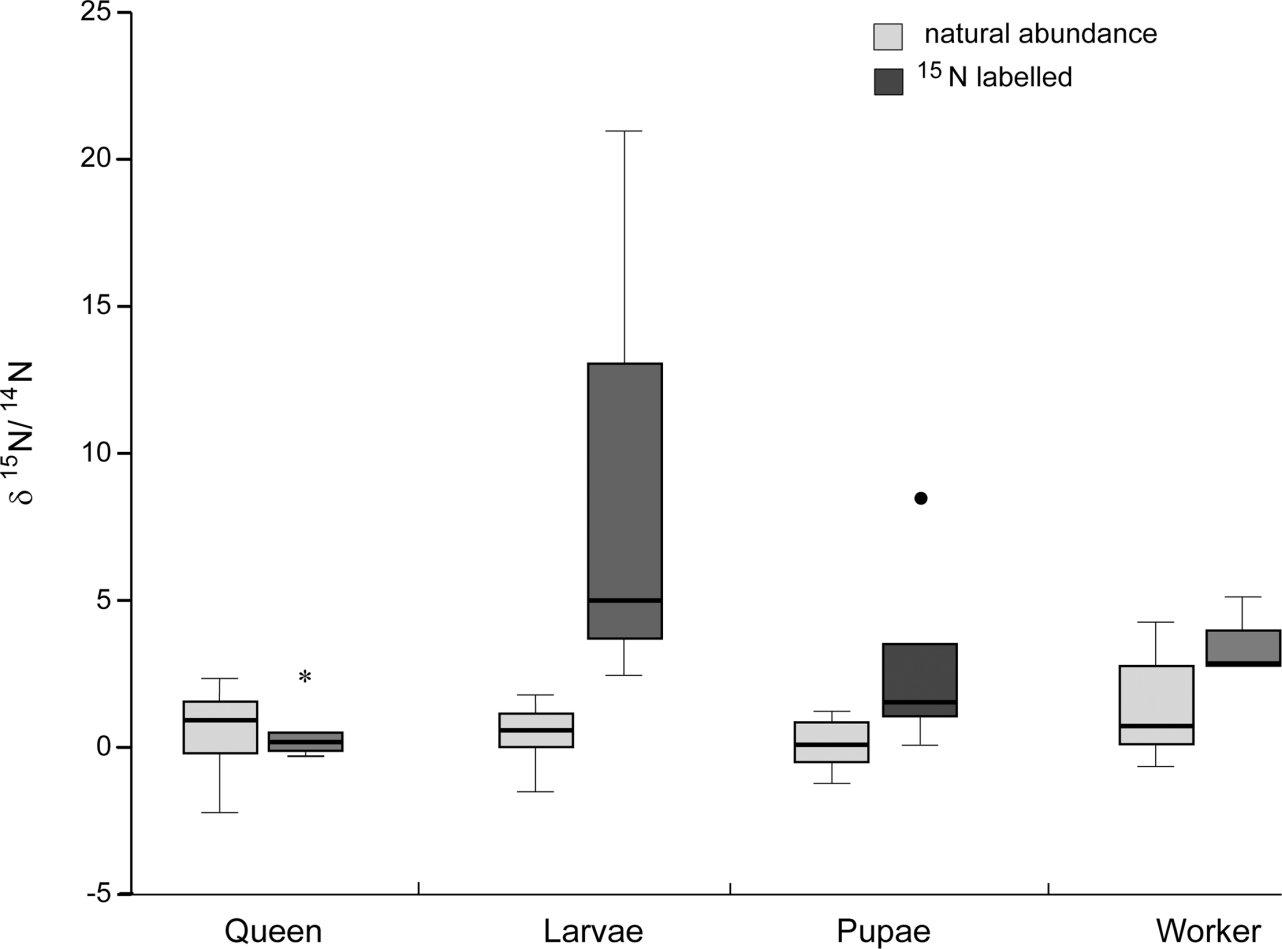
^15^N incubation of foundress patch. ^δ15^N values of founding queens, larvae and pupae after incubating the patches of the respective founding queen for 7 days with a ^15^N labelled amino acid mix. ^15^N accumulation could be shown for pupae and larvae, but only larvae were significantly enriched. The δ^15^N values of foundress queens from labelled patches did not differ from natural abundance. Hyphae were thus not eaten by the queens themselves but fed to the larvae.

The incubation of foundress patches with ^15^N amino acids led to a significant accumulation of ^15^N in larvae (Mann-Whitney-*U*-Test: *U* < 0.001, *p* = 0.004). Ant pupae and workers only showed a trend towards elevated ^15^N ratios, whereas queens did not accumulate any ^15^N (Fig 5, Table 3).

**Table 3:** Mean δ^15^N and C/N ratio of foundress queen patches from domatia with ^15^N amino acid mix labelled patches and unlabelled ones. SE gives the standard error of means, n the sample number size.

Sample	^15^N labelled					natural abundance				
	n_lab_	δ^15^N_label_	SE	C/N_lab_	SE	n_cont_	δ^15^N_cont_	SE	C/N_cont_	SE
patch	5	2115.029	679.51	37	5.6	13	137.311	38.083	48	13.3
queen	5	0000.449	0.50	10	2.3	05	000.463	00.790	16	04.0
larvae	4	0008.358	4.25	07	1.1	07	000.513	00.409	07	00.5
pupae	5	0002.998	1.52	07	0.8	07	000.121	00.353	07	00.6
worker	3	0003.590	0.78	05	0.6	05	001.372	00.903	05	00.3

## Discussion

Chaetothyrialean fungi as third party in numerous ant-plant mutualisms received increasing attention in the last years [5, 10, 11, 23, 24]. It was found that they are important for the ants’ nutrition and the system’s nutrient recycling [8, 25]. The present study is the first to focus on the establishment of the fungiculture at the beginning of a new colony cycle in an obligate ant-plant mutualism.

### The role of the domatium parenchyma

After entering the host-plant provided nesting space, *Azteca* foundress queens scrape parenchyma from the inner nest wall, seal the entrance and produce small piles. We could show that the parenchyma itself is free of chaetothyrialean fungi, only after contact with the ant queens, the parenchyma piles contain fungal hyphae. Parenchyma scraping and sealing of entrance holes with masticated plant tissue is known in other claustrally founding plant-ant queens [3, 40–42]. Whether parenchyma is also accumulated into a pile has, however, not been described in these earlier accounts. But the presence of chaetothyrialean fungi in these other systems was discovered recently [9–11] and the onset of fungiculture is likely similar among these different tropical fungus-cultivating plant-ants.

### Fungus transmission

As we did not find any hyphae in plant tissue of uninhabited domatia, we exclude an endophytic origin of the fungi in the parenchyma pile. Instead, vertical fungus transmission by the foundress queens seems plausible for the following reasons:

First, the broad overlap among genotypes and OTUs from established *Azteca* colonies (100% on OTU level, 82% on genotype level). Only two genotypes had not been found earlier in established colonies and three genotypes from established colonies [23] were not found in foundress patches (S1A Table). This is probably because we did not sample the respective mother colony or the respective reproductive ants.

Second, the overall diversity of chaetothyrialean genotypes/OTUs in the environment of the host plants may be at least ten times higher as shown by a study about chaetothyrialean fungi in carton galleries of *Azteca brevis* on the branches of various host trees [43]. This earlier study took place in one of the collection sites of the present study and resulted in 128 genotypes and 62 OTUs of black yeasts compared to only 14 genotypes from five OTUs in foundress queen patches. Due to this low OTU number in domatia, *de novo* recruitment of the fungi is highly unlikely.

Third, the chaetothyrialean lineage containing the domatia symbionts has so far been exclusively detected in domatia and is yet unknown from any other substrate, indicating a close association with their ant symbionts. Notably, there is no overlap with the carton galleries of *Azteca* mentioned above, which contains a high species biodiversity of various chaetothyrialean lineages but not from the domatia symbiont clade.

Fourth, the presence of hyphal parts and fungal spores in infrabuccal pockets of alate queens points to an inoculation of the parenchyma pile with fungal pellet material brought from the fungiculture of their mother colonies. Unfortunately, we were not able to generate DNA sequences from the infrabuccal pocket content making it uncertain whether the hyphal parts and spores found are truly those of the later domatia fungal community. A vertical transmission mode is also known from *Lasius fuliginosus* cultivating fungi on their nest walls [44], or from leaf cutter ants [45, 46] and ensures the occurrence of the right partners in the next generation of the mutualism. For chaetothyrialean fungi with their low competitive ability [11] ant nests may be an important ecological niche.

In addition to fungal parts and spores, dauer stages of nematodes were also carried by the foundress queens (Fig 4D). *Sclerorhabditis neotropicalis* (Rhabditida) is frequently found in *Cecropia* colonizing *Azteca* nests [47] but it was not known until now that *Sclerorhabditis neotropicalis* is vertically transmitted and already present during colony founding.

### Number and diversity of foundress queens and the range of fungal OTUs

Most of the foundress queen domatia showed haplometrotic colony founding with only one gyne present (76.6%). 86% of those single foundresses had parenchyma piles with one fungal OTU, 14% with more than one. In contrast, pleiometrotic colony founding was comparably rare, with multiple gynes of the same species in 12.8% and gynes from different ant species in 10.6% of the cases. Usually, in one *Cecropia* stem several independent founding events occur in the spatially segregated domatia. Multi-queen colonization of saplings and the survival of only one queen seems to be a common feature in ant-plant mutualisms and was described from several other ant-plant systems (e.g., *Crematogaster-Macaranga* [40]; *Tetraponera-Barteria* [42]; *Ocotea-Myrmelachista* [48]; *Triplaris-Pseudomyrmex* [49]. Also in *Cecropia* only one of the incipient colonies becomes dominant occupying all other domatia [50]and with a single queen only [51]. This may lead to the acquisition of the fungal patches from the less successful queens by the ant colony that wins the race and may explain why multi-OTU fungal patches occurred in 22% of the established *Cecropia* inhabiting *Azteca* colonies (Fig 3; Table 2). However, this leads to another question: why do 78% of the established colonies have only single-OTU fungal patches although multi-queen colonization of saplings is common with different OTUs in each foundress queen patch?

Two scenarios may be possible. (1) The gyne with the most vigorously growing fungal strain may be the first with enough workers to take over the stem and outcompete other colony foundings. The most vital fungus may overgrow their strains and become dominant, replacing the original fungi. (2) Directed symbiont selection could have happened with selection of the most vigorously growing strain. In *A*. *alfari*, for example, OTU2 and 3 were found in foundress queen patches, but in 96% of the established colonies only OTU2 was cultivated. The other OTU became under-represented and patches with multiple OTUs were rare with this ant species. *Azteca* ants probably groom their fungus patches as described for fungus-cultivating *Petalomyrmex phylax* living in domatia of *Leonardoxa africana* [9], or like leaf cutter ants do in their fungus gardens to control growth and microbial infections [52, 53]. Ants may not only be able to distinguish between pathogenic fungi and fungal symbionts but may also recognize the most beneficial fungal strain. A good example for such screening is the rejection of leaves harmful for fungal growth in leaf-cutter ant colonies. The ants perceive the state of the fungus through olfactory cues released from the fungus itself [54, 55].

### Chaetothyrialean fungi as food for the claustral queen?

Colony founding is a critical phase with high mortality rates: most ant queens with claustral colony founding deplete their flight muscles and fat deposits to maintain the metabolic needs and nurture their larvae, and most of them die from starvation [56–58] before the first workers emerge [49, 59, 60]. Therefore, various authors suggested that in *Cecropia* the foundress queen feeds on a nutrient rich callus tissue growing from the entrance hole or on the parenchyma of the inner domatia walls [61–63]. We did, however, not find a nutrient rich callus tissue in any of the domatia with young queens. Nor seemed the latex of the tissue plug used to seal the entrance hole to be suitable for queen nourishment and the fungal patch material was exclusively fed to the larvae. The availability of a fungal patch as food source may be a crucial factor for the development of the first workers–and indirectly for the survival of the queen as after depletion of her body’s own resources the queen is nurtured by the workers. The frequent occurrence of dead or moribund queens in very young *Cecropia* trees [64] may be due to the lack of a well-developed parenchyma layer, and abortive fungiculture. As a consequence, the larvae do not develop, no workers emerge, and the queen will die.

## Conclusion

We infer from our data, that in *Azteca/Cecropia* system the transmission of the fungal symbiont for fungiculture is vertical. Compared to the chaetothyriales found in the environment, the genetic variability of the fungi from fungal patches is limited indicating a selection of strains beneficial to the ant partner. That egg laying only occurs after establishing a fungal inoculum and the fact that larvae are nourished with patch material points to a fundamental importance of fungus cultivation for successful ant colony founding in this ant-plant system.

Agriculture has for a long time been regarded a cultural achievement of humans. In truth this uniqueness does not exist. Fungus farming is found in ants, beetles, termites and snails, [65, 66] many of them arose long before humans started farming. Fish and sloth have algal farms [67, 68], crabs and mussels bacteria gardens [69, 70].

Future work investigating mechanisms of fungus selection by the ants and the functional role of the ubiquitous fungal patches in ant-plant interactions in the tropics will help to increase our understanding of the richness both in species diversity and number of individuals of canopy ants in an in fact nutrient poor environment.

### Data accessibility

DNA sequences: Genbank accession numbers are MF066273-MF066356. Phylogenetic data, including alignments are available at TreeBASE, accession URL http://purl.org/phylo/treebase/phylows/study/TB2:S22034.

## Supporting information

S1 Table Foundress patches (n = 52) and patches of established colonies (n = 25) analyzed in this study, GenBank accession numbers, *Azteca* species, collection site (Monteverde, La Gamba), and genotype and OTU assignment. (DOC)

S2 Table Distribution of the foundress queens in the trees investigated. (DOCX)

S1 Fig Colonization of a young *Cecropia peltata* stem. (TIF)

S2 Fig Colony founding in *Cecropia* domatia. (TIF)

S3 Fig OTU frequency. (TIF)
