# Supplementary material for: Transmission of fungal partners to incipient Cecropia-tree ant colonies
Source: PLoS One. 2018 Feb 21;13(2):e0192207. doi: 10.1371/journal.pone.0192207 (PMC5821464; doi:10.1371/journal.pone.0192207)
Supplement: S1 Table — (DOC) [file pone.0192207.s001.doc]

**Supplementary Table S1a.** Foundress patches investigated (n=52), GenBank accession numbers, *Azteca* species, collection site (Monteverde, La Gamba), and genotype and OTU assignment. Patches with more than one genotype had Seq1, Seq2 etc additionally to the sample ID at the end. Genotype names refer to the Sequences defined as representative for the OTU (Nepel et al. 2016). Two genotypes are new (marked with an asterisk) and so far only known from foundress patches.

|  |  |  | |  | | OTU1 | | | OTU2 | | | | OTU3 | | OTU4 | OTU5 |
| --- | --- | --- | --- | --- | --- | --- | --- | --- | --- | --- | --- | --- | --- | --- | --- | --- |
| **Sample-ID** | **GenBank accession no** | | **Ant species** | **Collection site** | chaeD-CR-1 | | *chaeD-CR-13  **NEW** | chaeD-CR-2 | chaeD-CR-12 | chaeD-CR-5 | chaeD-CR-4 | *chaeD-CR-14  **NEW** | chaeD -CR-8 | chaeD-CR-7 | chaeD-CR-9 | chaeD-CR-11 |
| 13Cec7b | KX120996 | | *A. coeruleipennis* | Monteverde |  | |  |  | 1 |  |  |  |  |  |  |  |
| 13Cec7c | KX121007 | | *A. xanthochroa* | Monteverde | 1 | |  |  |  |  |  |  |  |  |  |  |
| 13Cec7d Seq1 | MF066300 | | *A. xanthochroa* | Monteverde | 1 | |  |  |  |  |  |  |  |  |  |  |
| 13Cec7d Seq2 | MF066301 | |  |  |  | |  |  |  | 1 |  |  |  |  |  |  |
| 13Cec7e | KX121013 | | *A. coeruleipennis* | Monteverde |  | |  |  |  |  |  |  |  |  | 1 |  |
| 13Cec7f Seq1 | MF066304 | | *A. constructor* | Monteverde | 1 | |  |  |  |  |  |  |  |  |  |  |
| 13Cec7f Seq2 | MF066302 | |  |  |  | |  |  |  |  |  |  | 1 |  |  |  |
| 13Cec7f Seq3 | MF066303 | |  |  |  | |  |  |  |  |  |  |  |  | 1 |  |
| 13Cec7g | MF066305 | | *A. constructor*/ *xanthochroa* | Monteverde | 1 | |  |  |  |  |  |  |  |  |  |  |
| 13Cec13I3 | MF066273 | | *A. constructor* | Monteverde | 1 | |  |  |  |  |  |  |  |  |  |  |
| 13Cec13I5 Seq1 | MF066274 | | *A. coeruleipennis* | Monteverde |  | |  |  |  |  |  |  | 1 |  |  |  |
| 13Cec13I5 Seq2 | MF066275 | |  |  |  | |  |  |  |  |  |  |  | 1 |  |  |
| 13Cec13I5 Seq3 | MF066276 | |  |  |  | |  |  |  |  | 1 |  |  |  |  |  |
| 13Cec13I5 Seq4 | MF066277 | |  |  |  | |  | 1 |  |  |  |  |  |  |  |  |
| 13Cec13I6_Seq1 | MF066278 | | *A. constructor* | Monteverde | 1 | |  |  |  |  |  |  |  |  |  |  |
| 13Cec13I6_Seq2  = *chaeD-CR-13 | MF066330 | |  |  |  | | 1 |  |  |  |  |  |  |  |  |  |
| 13Cec13I7 | KX120980 | | *A. constructor*/ *xanthochroa* | Monteverde |  | |  |  |  | 1 |  |  |  |  |  |  |
| 13Cec15Adetritus | MF066279 | | *A. constructor* | Monteverde |  | |  |  |  |  |  |  |  |  | 1 |  |
| 13Cec16I2 | MF066280 | | *A. xanthochroa* | Monteverde | 1 | |  |  |  |  |  |  |  |  |  |  |
| 13Cec16I3 | MF066281 | | *A. constructor* | Monteverde |  | |  |  |  |  |  |  |  | 1 |  |  |
| 13Cec16I5 | MF066282 | | *A. constructor* | Monteverde | 1 | |  |  |  |  |  |  |  |  |  |  |
| 13Cec19aI4  = *chaeD-CR-14 | MF066331 | | *A. constructor* | Monteverde |  | |  |  |  |  |  | 1 |  |  |  |  |
| 13Cec19aI5 FP1 | MF066283 | | *A. coeruleipennis* | Monteverde |  | |  |  |  | 1 |  |  |  |  |  |  |
| 13Cec19aI5 FP2 | MF066284 | | *A. constructor* |  |  | |  |  |  |  |  |  | 1 |  |  |  |
| 13Cec19cI5 | MF066287 | | *A. coeruleipennis*/ *constructor*/ *xanthochroa* | Monteverde |  | |  | 1 |  |  |  |  |  |  |  |  |
| 13Cec19aI6 | MF066285 | | *A. constructor* | Monteverde |  | |  |  |  |  |  |  |  |  | 1 |  |
| 13Cec19cI7 Seq1 | MF066288 | | *A. constructor*/ *xanthochroa* | Monteverde |  | |  | 1 |  |  |  |  |  |  |  |  |
| 13Cec19cI7 Seq2 | MF066289 | |  |  |  | |  |  |  |  |  |  |  |  | 1 |  |
| 13Cec19cI8 | MF066290 | | *A. constructor*/ *xanthochroa* |  |  | |  |  |  |  |  |  |  |  | 1 |  |
| 13Cec19aI9 | MF066286 | | *A. constructor* | Monteverde |  | |  |  |  |  |  |  |  | 1 |  |  |
| 13Cec19aI1 | KX120988 | | *A. constructor* | Monteverde |  | |  |  |  |  | 1 |  |  |  |  |  |
| 13Cec21domC Seq1 | MF066291 | | *A. constructor*/ *xanthochroa* | Monteverde | 1 | |  |  |  |  |  |  |  |  |  |  |
| 13Cec21domC Seq2 | MF066292 | |  |  |  | |  |  |  |  |  |  |  |  | 1 |  |
| 13Cec21domD Seq1 | MF066294 | | *A. coeruleipennis* | Monteverde |  | |  |  |  |  | 1 |  |  |  |  |  |
| 13Cec21domD Seq2 | MF066293 | |  |  |  | |  |  |  |  |  |  |  |  |  | 1 |
| 13Cec22domA | MF066295 | | *A. xanthochroa* | Monteverde | 1 | |  |  |  |  |  |  |  |  |  |  |
| 13Cec22domB Seq1 | MF066296 | | *A. coeruleipennis* | Monteverde | 1 | |  |  |  |  |  |  |  |  |  |  |
| 13Cec22domB Seq2 | MF066297 | |  |  |  | |  |  |  |  | 1 |  |  |  |  |  |
| 13Cec22domC | MF066298 | | *A. constructor*/ *xanthochroa* | Monteverde | 1 | |  |  |  |  |  |  |  |  |  |  |
| 13Cec23I3 | MF066299 | | *A. xanthochroa* | Monteverde | 1 | |  |  |  |  |  |  |  |  |  |  |
| 14Cec19i4 | MF066306 | | *A. alfari* | LaGamba |  | |  |  |  | 1 |  |  |  |  |  |  |
| 14Cec42i8 | MF066307 | | *A. alfari* */xanthochroa* | LaGamba |  | |  |  |  |  |  |  |  | 1 |  |  |
| 14Cec47i8 | MF066309 | | *A. xanthochroa* | LaGamba |  | |  |  |  |  |  |  |  | 1 |  |  |
| 14Cec47i10 | MF066308 | | *A. constructor* | LaGamba |  | |  |  |  |  |  |  |  | 1 |  |  |
| 14Cec8i3 | MF066325 | | *A. alfari* | LaGamba |  | |  |  |  |  |  |  |  | 1 |  |  |
| 14Cec82i3 | MF066311 | | *A. constructor* | LaGamba |  | |  |  |  |  |  |  |  | 1 |  |  |
| 14Cec82i13 | MF066310 | | *A. constructor* | LaGamba |  | |  |  |  |  |  |  |  | 1 |  |  |
| 14Cec84i6 | MF066312 | | *A. constructor* | LaGamba |  | |  |  |  |  |  |  |  | 1 |  |  |
| 15Cec9i4 | MF066326 | | *A. alfari* | LaGamba |  | |  |  |  | 1 |  |  |  |  |  |  |
| 15Cec10i14 | MF066313 | | *A. alfari* | LaGamba |  | |  |  |  | 1 |  |  |  |  |  |  |
| 15Cec11 | MF066314 | | *A. alfari* | LaGamba |  | |  |  |  | 1 |  |  |  |  |  |  |
| 15Cec19i12 | MF066317 | | *A. alfari* | LaGamba |  | |  |  |  |  |  |  |  |  | 1 |  |
| 15Cec26i7 | MF066318 | | *A. xanthochroa* | LaGamba |  | |  |  |  |  |  |  |  | 1 |  |  |
| 15Cec34i3i4 | MF066321 | | *A. alfari* | LaGamba |  | |  |  |  |  |  |  |  | 1 |  |  |
| 15Cec34i10 | MF066322 | | *A. xanthochroa* | LaGamba |  | |  |  |  |  |  |  |  | 1 |  |  |
| 15Cec34i11 | MF066323 | | *A. xanthochroa* | LaGamba |  | |  |  |  |  |  |  |  | 1 |  |  |
| 15Cec36i11 | MF066324 | | *A. alfari* | LaGamba |  | |  |  |  |  |  |  |  | 1 |  |  |
| 15CecPool1_2Fp | MF066327 | | *A. alfari* | LaGamba |  | |  |  |  |  |  |  |  | 1 |  |  |
| 15CecPool2_12Fp | MF066328 | | *A. alfari* | LaGamba |  | |  |  |  |  |  |  |  | 1 |  |  |
| 15CecPool3_3Fp | MF066329 | | *A. alfari* | LaGamba |  | |  |  |  | 1 |  |  |  |  |  |  |
| 15Cec15Pool 5Fp | MF066315 | | *A. alfari* | LaGamba |  | |  |  |  | 1 |  |  |  |  |  |  |
| 15Cec17Pool 2Fp | MF066316 | | *A. alfari* | LaGamba |  | |  |  |  | 1 |  |  |  |  |  |  |
| 15Cec30Pool 3Fp Seq1 | MF066319 | | *A. alfari* | La Gamba |  | |  |  |  | 1 |  |  |  |  |  |  |
| 15Cec30Pool 3Fp Seq2 | MF066320 | |  |  |  | |  |  |  |  |  |  |  | 1 |  |  |

**Supplementary Table S1b.** Patches of established colonies analyzed in this study (n=25), GenBank accession numbers, *Azteca* species, collection site, and genotype and OTU assignment. Genotype names refer to the sequences defined as representative for the OTU (Nepel et al. 2016).

|  |  | |  | OTU1 | OTU1 | | | OTU2 | | | | | OTU3 | | OTU4 | OTU5 |
| --- | --- | --- | --- | --- | --- | --- | --- | --- | --- | --- | --- | --- | --- | --- | --- | --- |
| **Sample-ID** | **GenBank accession no** | **Ant species** | | **Collection site** | chaeD-CR-1 | chaeD-CR-13 | chaeD-CR-2 | chaeD-FG-6 | chaeD-CR-12 | chaeD-CR-5 | chaeD-CR-4 | chaeD-CR-14 | chaeD-CR-8 | chaeD-CR-7 | chaeD-CR-9 | chaeD-CR-11 |
| 15Cec29i10_yc | MF066332 | *A. alfari* | | LaGamba |  |  |  |  |  | 1 |  |  |  |  |  |  |
| 15Cec33i5_yc_alf | MF066333 | *A. alfari* | | LaGamba |  |  |  |  |  |  |  |  |  | 1 |  |  |
| 15Cec42_EpI_II_alf | MF066334 | *A. alfari* | | LaGamba |  |  |  |  |  |  | 1 |  |  |  |  |  |
| 15Cec45_EcII_alf | MF066335 | *A. alfari* | | LaGamba |  |  |  |  |  | 1 |  |  |  |  |  |  |
| 16Cec1_EcII_const | MF066336 | *A. constructor* | | LaGamba |  |  |  |  |  | 1 |  |  |  |  |  |  |
| 16CecP2_EpIII_const | MF066337 | *A. constructor* | | LaGamba |  |  |  |  |  | 1 |  |  |  |  |  |  |
| 16CecP4_2EpII | MF066338 | *A. alfari* | | LaGamba |  |  |  | 1 |  |  |  |  |  |  |  |  |
| 16CecP5_EpIII | MF066339 | *A. constructor* | | LaGamba |  |  |  |  |  |  |  |  |  | 1 |  |  |
| 16CecP6_EpII_const | MF066340 | *A. constructor* | | LaGamba |  |  |  |  |  |  |  |  |  | 1 |  |  |
| 16Cec7_EcIII_alf | MF066341 | *A. alfari* | | LaGamba |  |  |  |  |  |  |  |  |  | 1 |  |  |
| 16Cec8_EpI_alf | MF066342 | *A. alfari* | | LaGamba |  |  |  |  |  | 1 |  |  |  |  |  |  |
| 16Cec10_EpI_alf | MF066343 | *A. alfari* | | LaGamba |  |  |  |  |  | 1 |  |  |  |  |  |  |
| 16Cec11_EpIII_const | MF066344 | *A. constructor* | | LaGamba |  |  |  |  |  | 1 |  |  |  |  |  |  |
| 16Cec13_EcII_const | MF066345 | *A. constructor* | | LaGamba |  |  |  |  |  |  |  |  |  | 1 |  |  |
| 16Cec14_EpIII_alf | MF066346 | *A. alfari* | | LaGamba |  |  |  |  |  | 1 |  |  |  |  |  |  |
| 16Cec16_EpIII_const | MF066347 | *A. constructor* | | LaGamba |  |  |  |  |  |  |  |  |  | 1 |  |  |
| 16Cec17_EpIII_alf | MF066348 | *A. alfari* | | LaGamba |  |  |  |  |  | 1 |  |  |  |  |  |  |
| 16Cec19 EcIII_const | MF066349 | *A. constructor* | | LaGamba |  |  |  |  |  |  |  |  |  | 1 |  |  |
| 16Cec20_EpIII_const | MF066350 | *A. constructor* | | LaGamba |  |  |  |  |  |  |  |  |  | 1 |  |  |
| 16Cec21_EcIII_alf | MF066351 | *A. alfari* | | LaGamba | 1 |  |  |  |  |  |  |  |  |  |  |  |
| 16Cec23_EpII_const | MF066352 | *A. constructor* | | LaGamba |  |  |  |  |  |  |  |  |  | 1 |  |  |
| 16Cec24_EpIII_const | MF066353 | *A. constructor* | | LaGamba |  |  |  |  |  |  |  |  |  | 1 |  |  |
| 16Cec25_EpIII_const | MF066354 | *A. constructor* | | LaGamba |  |  |  |  |  |  |  |  |  | 1 |  |  |
| 16Cec26_EcII_const | MF066355 | *A. constructor* | | LaGamba |  |  |  |  |  |  |  |  |  | 1 |  |  |
| 16Cec27_EpII_const | MF066356 | *A. constructor* | | La Gamba |  |  |  |  |  |  |  |  |  | 1 |  |  |
