# Supplementary material for: Transmission of fungal partners to incipient Cecropia-tree ant colonies
Source: PLoS One. 2018 Feb 21;13(2):e0192207. doi: 10.1371/journal.pone.0192207 (PMC5821464; doi:10.1371/journal.pone.0192207)
Supplement: S2 Table — (DOCX) [file pone.0192207.s002.docx]

**Supplementary Table S2. Distribution of the foundress queens in the trees investigated.** Total number of available domatia, the actually inhabited ones and the number of queens per domatium is given.

| **Site** | **year** | **no trees** | **no domatia** | **occupied dom** | **no queens alive** | **no queens dead** | **average/plant** | **plants with pleometrosis** |
| --- | --- | --- | --- | --- | --- | --- | --- | --- |
| La Gamba | 2012 | 17 | 283 | 34 | 36 | 2 | 2,1 | 1 |
| Monteverde | 2013 | 11 | 104 | 69 | 96 | 12 | 6,7 | 15 |
| La Gamba | 2014 | 12 | 118 | 15 | 17 | 1 | 1,4 | 2 |
| La Gamba | 2015 | 24 | 222 | 62 | 63 | 10 | 2,2 | 2 |
| **Total** |  | 64 | 727 | 180 | 212 | 25 | 3,7 | 20 |
