# Supplementary figures and images for: Transmission of fungal partners to incipient Cecropia-tree ant colonies
Source: PLoS One. 2018 Feb 21;13(2):e0192207. doi: 10.1371/journal.pone.0192207 (PMC5821464; doi:10.1371/journal.pone.0192207)

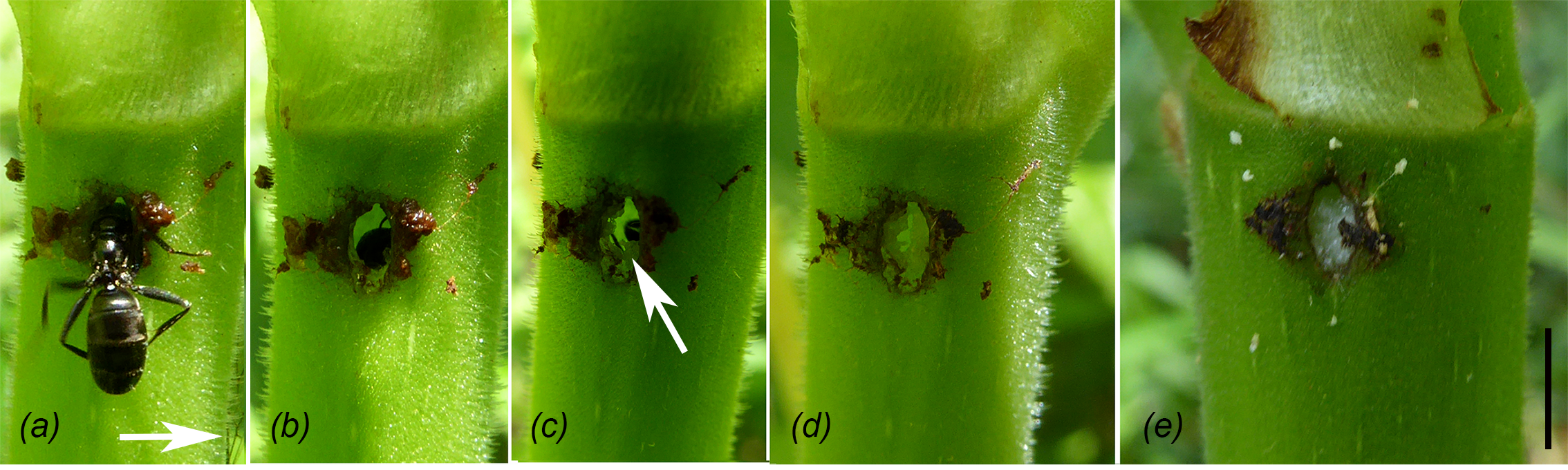

Supplement: S1 Fig — (TIF) [file pone.0192207.s003.tif]

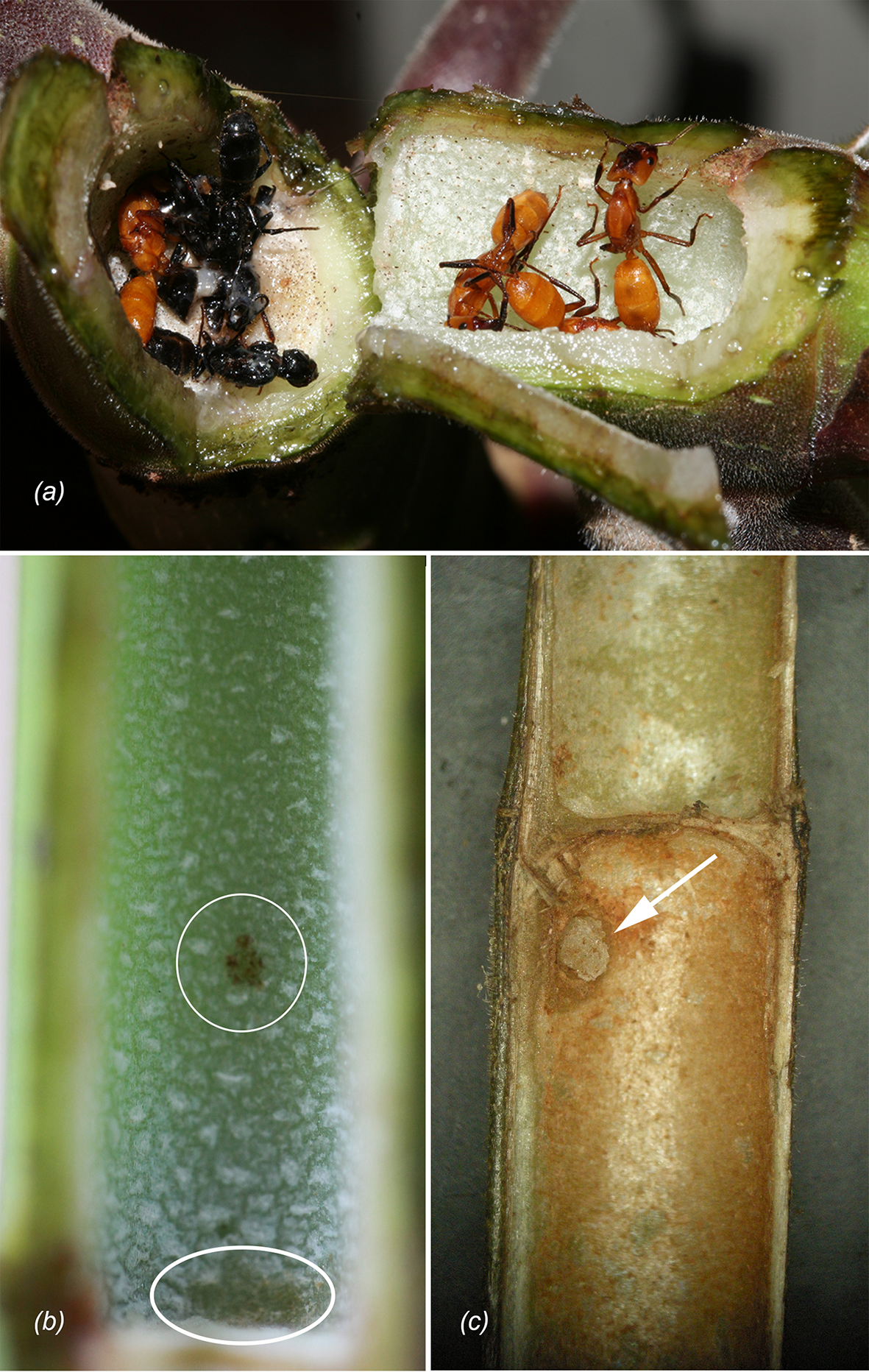

Supplement: S2 Fig — (TIF) [file pone.0192207.s004.tif]

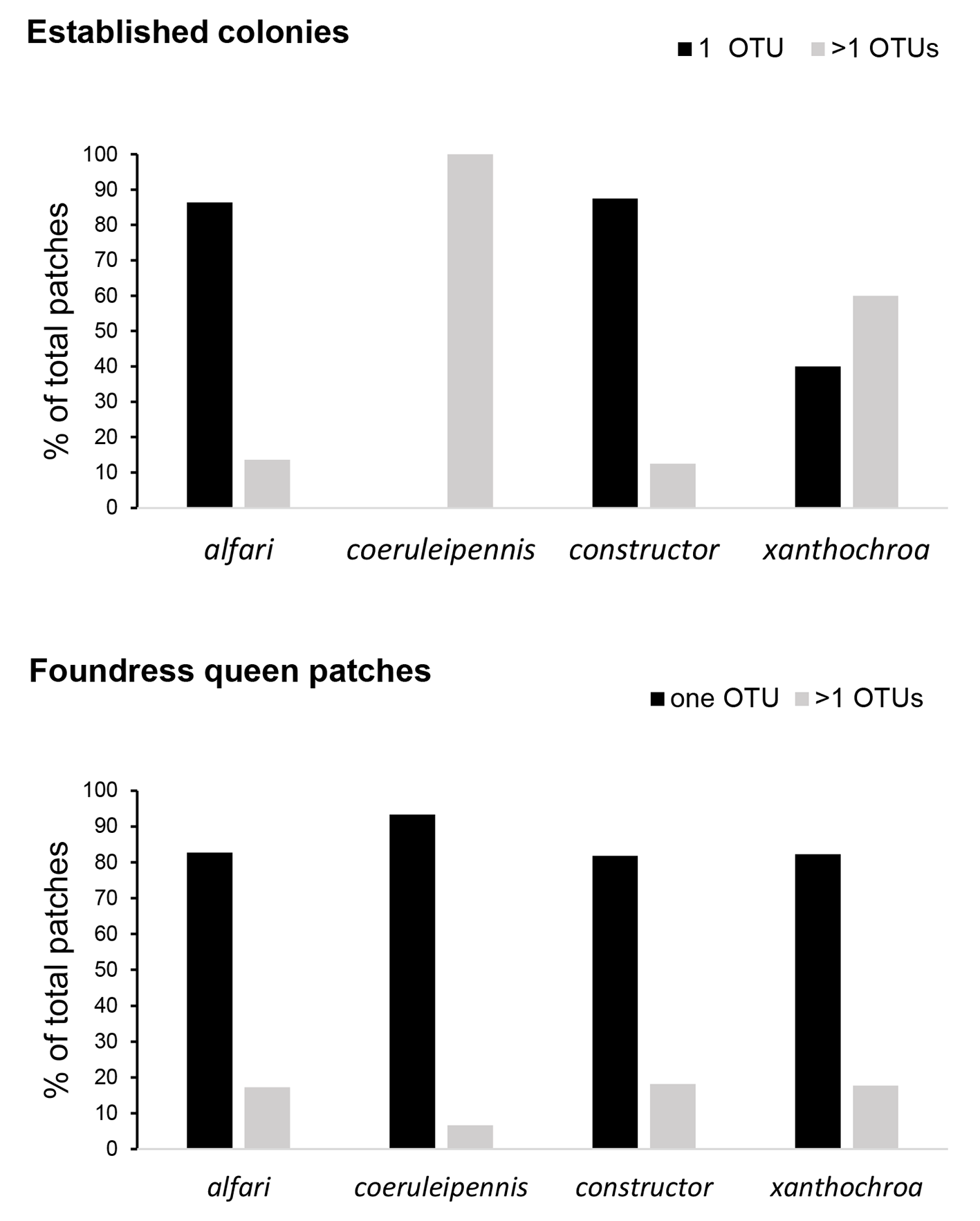

Supplement: S3 Fig — (TIF) [file pone.0192207.s005.tif]
